# The Effects of Brain Breaks on Motives of Participation in Physical Activity among Primary School Children in Malaysia

**DOI:** 10.3390/ijerph16132331

**Published:** 2019-07-02

**Authors:** Mawar Siti Hajar, Hussein Rizal, Yee Cheng Kueh, Ayu Suzailiana Muhamad, Garry Kuan

**Affiliations:** 1 Exercise and Sport Science Programme, School of Health Sciences, Universiti Sains Malaysia, Kelantan 16150, Malaysia; 2 Unit of Biostatistics and Research Methodology, School of Medical Sciences, Universiti Sains Malaysia, Kelantan 16150, Malaysia; 3 Department of Life Sciences, Brunel University, London UB8 3PH, UK

**Keywords:** Brain Breaks^®^, physical activity, motivation, children, primary school

## Abstract

Brain breaks is a physical activity (PA) video designed for school settings that is used to stimulate student’s health and learning. The purpose of this study is to measure the effects of brain breaks on motives of participation in PA among primary school children in Malaysia. Purposive sampling was used to divide 159 male and 176 female students aged 10 to 11 years old, mean (standard deviation (SD)) = 10.51 (0.50), from two schools in Kelantan, Malaysia into intervention (*n* = 183) and control (*n* = 152) groups. Students undertook brain breaks activities on school days (five minutes per session) spread out for a period of four months. Mixed factorial analysis of variance (ANOVA) was used to test the students’ motives of participation in PA for pre-, mid-, and post-tests using the Physical Activity and Leisure Motivation Scale-Youth-Malay (PALMS-Y-M). Mixed factorial ANOVA showed significant changes in enjoyment, *F*(2, 392) = 8.720, *p*-value (η_p_^2^) = 0.001 (0.043); competitiveness, *F*(2, 195) = 4.364, *p*-value (η_p_^2^) = 0.014 (0.043); appearance, *F*(2, 392) = 5.709, *p*-value (η_p_^2^) = 0.004 (0.028); and psychological condition, *F*(2, 392) = 4.376, *p*-value (η_p_^2^) = 0.013 (0.022), whereas mastery, affiliation, and physical condition were not significant (*p* < 0.05). Further post-hoc analysis revealed a significant downward trend in the control group (*p* < 0.05). Brain breaks is successful in maintaining students’ motives for PA in four of the seven factors.

## 1. Introduction

The relationship between regular physical activity (PA) and psychological and physical health has been thoroughly established [1]. The takeaway message is that regular PA provides essential health benefits, one of which is the reduction of non-communicable diseases and the reduced rate of mortality [2,3]. In addition, regular PA could contribute in increased mental health and academic performance, and lower stress and depression levels [4], as well as demonstrate improvement in math skills, cognitive flexibility, improved memory, and creativity when engaged in sufficient PA such as aerobic exercise [5,6].

Nonetheless, in Malaysia, people are not active enough to take advantage of the health benefits provided by regular PA; instead they tend to lead a sedentary lifestyle [7]. Furthermore, individuals who were physically inactive during adolescence are more likely to be inactive in their adulthood [8]. For these reasons, researchers, health professionals, and policymakers have all sought to explore why some people are physically active, whereas others are not [9]. Although the factors for participating in regular PA are highly complex, studies have pointed out that motivation is a driving factor not only for PA participation but also for its adherence [10,11].

Research had demonstrated the link between motivation and PA [12]. Researchers have used motivational theories such as self-determination theory [13] and achievement goal theory [14] to act as guidelines in studying human motivation in PA. For example, in the self-determination theory, individuals who are intrinsically motivated to undertake PA are motivated by factors including enjoyment, challenge, skill development, and mastery [15,16], whereas individuals who are extrinsically motivated to undertake PA are motivated by factors including rewards, improved health, and appearance that are not related to the activity itself [15,16]. Hence, understanding people’s motives for participation in PA is crucial, given its role in determining whether individuals will initiate and maintain PA programs [17].

One such tool that can be used to measure the motives for participating in PA is the Recreational Exercise Motivation Measure (REMM) that was developed by Rogers and Morris [18]. Due to the sizable length of the questionnaire (73 items), a newer version called the Physical Activity and Leisure Motivation Scale (PALMS) [18] was created. It was designed for adults and contained 40 items divided into eight separate factors: enjoyment, mastery, competition/ego, appearance, affiliation, others’ expectations, psychological condition, and physical condition. For children, Hu et al. [19] shortened the scale to 28 items comprised of seven factors, removing others’ expectation and reducing the least strong item from each motive factor to produce a shorter scale suitable for younger people (PALMS-Y). The scale was later translated and validated using a confirmatory factor analysis into the Malay language (PALMS-Y-M), and the results indicated sound validity and reliability [20].

The low and reducing prevalence of PA in younger people is of particular concern [6,19]. It is suggested that the ideal interventional strategy for PA promotion that is cost-effective is a school-based intervention for children and adolescents [21]. The school environment is ideal for implementing PA interventions due to the possibility of reaching a vast number of children who are spending most of their time in schools [22]. Presently, implementation of school-based PA programs indicated positive improvements in cognitive skills and attitudes, academic performance, and academic behaviour [23]. However, more research is required to investigate the effects of school-based PA to support the effort of initiating policies for promoting positive changes at the decision-making levels aimed at providing children with more regular access to PA in school settings [24].

One such intervention is brain breaks, a web-based structured PA break that stimulates student’s health and learning; and is specifically designed for the classroom setting [24]. This PA intervention, which was introduced by HOPSports, is used to motivate students to enhance their theoretical lessons and provide an opportunity for them to not only be physically active during breaks but also for them to learn new motor skills such as language, art, music, and different cultures [25]. It is part of a Global Community Health project, supported by the Centers for Disease Control and Prevention, and it involves the whole school, community, and child framework [26].

The program is supported by the United Nations as part of the 17 Sustainable Developmental Goals under the goals of good health and well-being [27]. To access the resource (https://brain breaks.com/), users only require an internet connection and a projector to display the PA videos. More studies are needed in identifying the motives of younger people in participating and/or abstaining from PA in order to alter the increasing trend of physical inactivity among youths in many countries [17]. Collaboration between researchers and schools are needed to provide PA to the students in between or during the class sessions.

Past studies have used the Attitudes toward Physical Activity Scale (APAS) [24,28,29,30] to measure children’s’ attitudes and perceptions regarding various aspects of engagement in PA, including brain breaks [29]. These studies have shown positive findings in terms of promoting PA among school children [24,28,29,30]. The understanding of motivated behavior of adolescence and young adults is essential to encourage their persistence in sports and to increase their PA participation. This will positively contribute to the development of their physical and psychological well-being [31]. A previous study examined the REMM motives for taking part in recreational exercise/sporting activities [32]. However, to our knowledge, studies about PALMS are limited, and no studies using PALMS-Y-M as a measure of motivation in a longitudinal study were found. Therefore, this study attempts to identify the motives for PA, particularly among Malaysian children, while using brain breaks as a means of PA intervention. The hypothesis is that brain breaks has a significant effect on PA motives, particularly enjoyment, competition, mastery, affiliation, appearance, as well as physical and psychological conditions.

## 2. Participants and Methods

### 2.1. Study Design, Recruitment, and Sampling

This study employed a quasi-experimental design. Initially, simple randomization was used to recruit the schools, however, as one of the schools selected was a high performance school (excellent award school), the authors switched to a purposive sampling to avoid disparity in academic achievement. By using purposive sampling, two high performance schools in the state of Kelantan were selected, in which 16 classes from standards four and five were chosen. The students were divided into two groups: intervention and control groups. All the students in the recruited classes were invited to participate. Students with prior injuries or conditions such as heart problems were excluded. Students who were currently engaged in long-term school activities that hindered consistent participation or had not acquired parental consent were also excluded.

### 2.2. Participants

The total sample size was 335 students, with 183 students in the intervention group and 152 students in the control group. The descriptive data of the students are presented in Table 1 in which the age is presented in mean (standard deviation; SD), whereas, the gender and group are presented in frequency (%). Means (SD) were analysed by using descriptive statistics.

### 2.3. Procedures

The study received approval from the Universiti Sains Malaysia (USM) Human Research Ethics Committee (USM/JEPeM/18020104) and was conducted in accordance with the guidelines of the International Declaration of Helsinki. Approval from the Ministry of Education and the State Education Department were also acquired.

Two schools were purposively selected and split into the intervention and control groups. Only the intervention group went through four months of brain breaks intervention (including examination week, school activities, and school holidays). Students and teachers from both schools were briefed on the objectives and procedures of the study. All students from both groups had to take a pre-test by completing the PALMS-Y-M questionnaire. Following that, students in the intervention group participated in the brain breaks activities, five minutes, five times per week, spread out for a period of four months, similar to previous brain breaks studies [24,28,29,30]. Students in the control group were not involved with the brain-breaks activity. Instead, they were asked to follow their normal class schedule.

The brain breaks video was projected on a screen using a projector at the school hall. A new brain breaks video was consistently uploaded and shown to the students to maintain enjoyment and motivation. Students’ movement varied according to the brain breaks video; therefore, precautions were taken by making sure enough space was given for the students to move during the session. Online access to the official project website is found at http://hopsports.com/what-is-brain-breaks [29]. The PALMS-Y-M questionnaires were administered for pre-, mid-, and post-test in both groups. Each student was given a unique code to match them across the test sessions and to maintain confidentiality.

### 2.4. Physical Activity and Leisure Motivation Scale-Youth-Malay (PALMS-Y-M)

The PALMS-Y-M consists of 28 items with seven factors measuring different types of motives for participating in physical and leisure activities [17] including enjoyment, mastery, competition and ego, appearance, affiliation, physical condition, and psychological condition. Examples of items of each respective factors are “to get better at an activity” (mastery), “because it’s interesting” (enjoyment), “to better cope with stress” (psychological condition), “because it helps maintain a healthy body” (physical condition), “to define muscle, look better” (appearance), “to do activity with others” (affiliation), and “because I perform better than others” (competition/ego). The scale is a 5-point Likert scale from 1 (*strongly disagree*) to 5 (*strongly agree*), whereby a higher score reflects that participants experience a higher level of that motive for participating in physical and leisure activities [17]. This scale has been previously validated by Kueh, et al. [20] in a Malaysia-based sample, in which the internal consistency for each motive factor based on Cronbach’s Alpha was as follows: 0.77 (enjoyment), 0.77 (mastery), 0.76 (competition/ego), 0.73 (affiliation), 0.85 (appearance), 0.79 (physical condition), and 0.83 (psychological condition).

### 2.5. Data Analysis

The analysis for this study was conducted using the Statistical Package for Social Science (SPSS) Version 24.0 (IBM, Armonk, NY, USA). Mixed factorial analysis of variance (ANOVA) with time as the within-subject factor and group (experimental vs. control) as the between-subject factor was used to analyze the effects of brain breaks intervention on the PALMS-Y-M factors. In addition, Bonferroni correction was used to measure the differences between time points (post-hoc analysis). Cohen’s classification of effect size with equivalent values of eta squared (0.01 ≤ eta^2^ < 0.06 = small, 0.06 ≤ eta^2^ < 0.14 = medium, eta^2^ > 0.14 = large was referred [33]. Results were considered significant at *p* < 0.05.

## 3. Results

Table 2 shows the mean (SD) for each motive factor found in PALMS-Y-M for each test. Significant findings were then calculated using mixed factorial ANOVA for each motive subscale (Table 3). Post-hoc analysis for subsequent significant results are tabulated in Table 4. Mixed factorial ANOVA for mastery, affiliation, and physical condition revealed no significant changes. Therefore, no further post-hoc analyses were conducted for these factors.

For the enjoyment factor, post-hoc analysis revealed changes in the control group across tests: pre- to mid-tests (*p*-value = 0.018), pre- to post-tests (*p*-value = 0.001), and mid- to post-tests (*p*-value = 0.022). Post-hoc analysis for the competition factor showed significant changes in the control group for pre- to mid-tests (*p*-value = 0.018), pre- to post-tests (*p*-value = 0.001), and mid- to post-tests (*p*-value = 0.022). Appearance factor showed significant changes with post-hoc analysis in the control group for pre- to mid-tests (*p*-value = 0.024) and pre- to post-tests (*p*-value = 0.001). For the psychological condition factor, post-hoc analysis showed significant changes for the intervention group for pre- to mid-tests (*p*-value = 0.026). Whereas, in the control group, significant changes were found for pre- to post-tests (*p*-value = 0.013) and mid- to post-tests (*p*-value = 0.027).

## 4. Discussion

The present study evaluates the effects of the brain break video exercise on motives of participation in PA. PALMS-Y-M measures seven motives of participation in PA: enjoyment, mastery, competition, affiliation, appearance, physical condition, and psychological condition. As seen in the results, four of the constructs showed a significant time effect, which included enjoyment and competition, as well as appearance and psychological condition. In addition, the enjoyment factor also showed significant results for the group effect, where the mean of the enjoyment score was found to be significantly higher in the control group compared to the intervention group (Table 3). However, the mean score of the enjoyment factor in the control group was already high at the beginning of the study (pre-test).

Based on further inspection on the mean trends, the intervention group showed either no discernible trend or non-significant upward trend (*p* < 0.05) except for the psychological condition, in which the mean difference from pre- to mid-tests showed a significant increase (*p* = 0.026), albeit not throughout the intervention period. Interestingly, however, there was a downward trend of all four constructs across the intervention period (pre- to post-tests) in the control group: enjoyment (*p* = 0.001), competition (*p* = 0.001), appearance (*p* = 0.001), and psychological condition (*p* = 0.013). These results indicate that brain breaks might minimise the reduction of motives for participation in PA among children over time.

A growing number of studies show a trend of decreasing PA level among children [30,34,35]. More worryingly, PA has also been found to decline with age [34,35]. A systematic review by Allender et al. [36] concluded that the motivations for PA among children are experimentation, unusual activities, parental support, and a safe environment. Participation for children was found to be more enjoyable when they were not being forced to compete and win but encouraged to experiment with different activities [36]. As children grow older, their motivations to be more physically active evolve to suit their current needs such as maintaining their body shape, weight management, creating new social networks, family support and peer support for adolescents, sense of achievement, skill development, medical sanction, health benefits, and enjoyment for adults [37,38,39,40,41]. The motives for PA for children in this study did not indicate any particular trend. Instead, they remained relatively stable across the intervention period.

As the psychological condition was the only trend that showed an increase in the intervention group, children’s maintenance in enjoyment, competitiveness, appearance and psychological condition should be more emphasized when compared with the control group. For children and youth, Biddle and Mutrie [42] reported that the motives for participation in PA were fun, skill development, affiliation, fitness, success, and challenge, whereas for adults, the motives change across stages of their lifecycles. Younger adults are motivated more by challenge, skill development, and fitness, whereas older adults are more interested in participation for reasons of health, relaxation, and enjoyment [42]. Ebben and Brudzynski [43] further emphasised that general health and maintaining fitness were the two most common themes for young adults. Teachers should then keep in mind the motives for PA of children when planning any PA session with children and adapting existing curricular activities to suit the psychological needs of the students. For example, there should be a focus on placing emphasis on enjoyment and competitiveness when planning a PA with the students.

The use of REMM has revealed that the motivation factors were the most often cited reasons by adults, which included physical fitness, psychological state, and enjoyment [10,44]. Similarly, the present study suggests that children may maintain both their enjoyment and psychological condition when introduced to more PA. However, conflicting with previous studies, the physical condition was not significant in either group. Health seems to be the most important motivation factor, regardless of age, gender, or level of PA, for participating in PA [32,45,46]. Active people often rated health, stress management, and enjoyment as more meaningful motivation factors compared to inactive people [10,45]. The present study made no distinction between active and inactive participants, which may be a defining factor in the non-significance of the intervention group. The previous longitudinal study indicated that environmental influences on leisure-time PA increase with age, which in turn, may influence the motives [47].

Past research has shown that motivation for participation also varies across different cultures [48,49,50]. The emphasis on a healthy lifestyle is not as prevalent in some global cultures as it is in the Western world, in which cultural perspectives can also vary on what is considered to represent an attractive appearance and the extent to which it is considered appropriate or important to commit time and effort to improve appearance [51]. For example, in England, the most important motivational factors for PA were ‘to physically feel in good shape, ‘to improve or maintain health’, and ‘to feel a sense of achievement’ [42]. Whereas, in Turkey, the most important participation motives were for health, followed by enjoyment, while the competition motive was the least important participation motive [32]. Hence, cultural differences should be considered before interpreting the PALMS questionnaire [48,49,50].

Besides cultural difference, gender may have influenced the result of this study. Past studies indicated significant gender differences on specific subscales of the questionnaire. As anticipated, females rated appearance as the primary motive for engaging in PA [52]. This is consistent with previous research [53,54] and it reflects an extrinsic motivation orientation that might be attributed to gender role socialisation processes and pressures to be slim and fit. In this study, appearance was not significant in the intervention group. However, it was a contributing factor in the downward trend in the control group. In addition, during the brain breaks session, males were seen to be more enthusiastic and motivated to follow the movements in the video, whereas, females were seen to be more sluggish and less inclined to participate, especially in more vigorous sessions. Nonetheless, they responded well when pushed by the teachers and found more enjoyment when participating in traditional dance especially when related to their culture (i.e., Malay dance). Therefore, researchers may specify the overall exercise load given by implementing less vigorous brain breaks to ensure equal participation from both females and males.

The difference in the level of PA participation and sports was also not considered in this study; a previous study showed that in team sports, players rated affiliation higher than the rest of the sample [52]. Besides, as shown in previous studies [16,55], gym-based exercisers rated physical health and appearance as more important, while martial arts (taekwondo) players and individuals engaging in yoga rated psychological health and mastery as the principal motives for engaging in PA. The present study showed no significance in regard to affiliation as the students were recruited from a highly academic oriented school; thus, not much emphasis has been placed on sports to begin with. PA has been demonstrated in studies to increase blood flow to the brain with an increase in oxygen level, which has the potential to have an impact on brain function [29]. The present study showed no significance for physical condition (let alone cognitive improvements) as more time can be better spent on academically oriented tasks.

The gym-based exercisers, on the other hand, seem to be more interested in enhancing their looks and maintaining a good physique, so it is not surprising that they rated physical health and appearance as their primary motives for engaging in PA [52]. Both physical condition and appearance were not significant in the present study. Whereas martial arts (taekwondo) players and individuals engaging in yoga were found to be more interested in improving their overall mental state and skill level. These findings suggested that team sports participants place more emphasis on the communal reasons for engaging in PA than the rest of the sample. These students were more interested in enjoying the social benefits of sports participation [52]. As the students in the present study are not sports-oriented, thus, no significance was found for both appearance and physical condition in the intervention group.

Previous uses of PALMS in an interventional study in a younger population are limited and therefore require more research. The use of brain breaks as a PA intervention is proven as an effective method in improving PA among children. Therefore, it should be assessed using other measuring tools such as the transtheoretical model [56,57] and APAS [24,28,29,30]. In addition, breaks during a lesson have also had a positive impact on learners’ motivation and achievement and on the student’s enjoyment during learning, their motivation for learning, and their focus [58]. Other implementations of the brain breaks are simple transitional physical and mental exercises designed to equip the teacher with the tools to manage the physiology and attention of the class as well as to keep the children in the most receptive state for learning [59]. Some limitations should be noted, such as the self-reporting nature of the surveys and the strict curricula and limited time to implement brain breaks during school hours. In addition, there was no use of an objective measure such as an accelerometer during the sessions, which would have made the study as a whole stronger. With that said, the present study was based on a real-world study occurring in a real-world setting, which is the strength of this study.

## 5. Conclusions

In conclusion, brains breaks effectively helped to maintain the enjoyment of participating in PA over time, especially when it is performed together with peers as it helps to trigger a healthy competition. In addition, appearance, competition, and psychological condition were maintained in the intervention group, whereas in the control, a significant downward trend was seen throughout the same period. Brain breaks shows evidence as a counter-measure to reduce and/or prevent losses in motives of participation in PA. Furthermore, PALMS-Y-M is an effective scale for measuring motives towards PA, primarily enjoyment, competition, appearance, and psychological condition. As suggestions for future studies, researchers should consider the cultural and gender differences, especially among the Muslim community and female students as it may affect their motives and willingness to participate in PA. Considering the cultural and gender differences of the targeted community is important to obtain more holistic and effective results for the entire sample.

## Figures and Tables

**Table 1:** Descriptive data of the participants (*n* = 335). SD = standard deviation.

Demographic Information	Mean (SD)	Frequency (%)
Age	10.51 (0.50)	
Gender:		
Male		159 (47.5)
Female		176 (52.5)
Groups:		
Intervention		183 (54.6)
Control		152 (45.4)

**Table 2:** Mean (SD) for each motive subscale.

Motive Subscales	Mean (SD)					
	Pre-Test		Mid-Test		Post-Test	
	Int ^†^	Con ^‡^	Int ^†^	Con ^‡^	Int ^†^	Con ^‡^
Enjoyment	12.29 (3.24)	13.93 (4.37)	12.28 (3.18)	13.04 (4.08)	11.81 (3.06)	12.18 (4.19)
Mastery	12.31 (2.98)	12.85 (3.42)	12.10 (3.01)	12.72 (3.54)	12.29 (2.66)	12.65 (3.62)
Competition	10.65 (3.37)	11.36 (3.63)	10.84 (2.94)	11.88 (3.46)	11.98 (3.24)	11.80 (3.59)
Affiliation	11.63 (3.12)	12.23 (3.66)	11.75 (2.92)	12.35 (2.88)	12.15 (2.96)	12.02 (3.20)
Appearance	13.64 (3.64)	14.92 (3.51)	13.77 (3.08)	14.06 (3.57)	13.93 (2.99)	13.44 (3.57)
Physical	12.79 (3.76)	13.45 (3.56)	13.31 (3.31)	13.06 (3.50)	12.82 (3.42)	13.06 (3.51)
Psychological	12.18 (3.37)	13.12 (4.02)	13.03 (3.02)	12.90 (3.41)	12.73 (2.81)	12.07 (3.41)

^†^ Intervention, ^‡^ Control.

**Table 3:** Repeated measure of variance, *F*-statistics, and main effects for each motive subscale.

Motive Subscales	*F* (df), *p*-value (Partial Eta^2^)		
	Group	Time	Time * Group
Enjoyment	4.652 (1, 196), 0.032 * (0.023)	8.720 (2, 392), 0.001 * (0.043)	2.923 (2, 392), 0.055 (0.015)
Mastery	2.041 (1, 196), 0.155 (0.010)	0.261 (2, 195), 0.771 (0.003)	0.141 (2, 195), 0.869 (0.001)
Competition	2.064 (1, 196), 0.152 (0.010)	4.364 (2, 195), 0.014 *(0.043)	2.911 (2, 195), 0.057 (0.029)
Affiliation	1.251 (1, 196), 0.265 (0.006)	0.177 (2, 392), 0.838 (0.001)	1.233 (2, 392), 0.292 (0.006)
Appearance	0.910 (1, 196), 0.341 (0.005)	2.611 (2, 392), 0.075 (0.013)	5.709 (2, 392), 0.004 * (0.028)
Physical	0.321 (1, 196), 0.572 (0.002)	0.419 (2, 392), 0.658 (0.002)	1.346 (2, 392), 0.261 (0.007)
Psychological	0.020 (1, 196), 0.887 (0.001)	2.094 (2, 392), 0.125 (0.011)	4.376 (2, 392), 0.013 * (0.022)

* Significant difference (*p* < 0.05).

**Table 4:** Post-hoc for motive subscales of enjoyment, competition, appearance, and psychological condition.

Groups	Tests	*p*-value			
		Enjoyment	Competition	Appearance	Psychological
Intervention	Pre-mid Pre-post Mid-post	0.978 0.225 0.201	0.978 0.225 0.201	0.728 0.438 0.658	0.026 * 0.188 0.414
Control	Pre-mid Pre-post Mid-post	0.018 * 0.001 * 0.022 *	0.018 * 0.001 * 0.022 *	0.024 ^*^ 0.001 ^*^ 0.089	0.559 0.013 * 0.027 *

* Significant difference (*p* < 0.05).
